# Effect of initial calorie intake via enteral nutrition in critical illness: a meta-analysis of randomised controlled trials

**DOI:** 10.1186/s13054-015-0902-0

**Published:** 2015-04-20

**Authors:** Feng Tian, Xinying Wang, Xuejin Gao, Xiao Wan, Chao Wu, Li Zhang, Ning Li, Jieshou Li

**Affiliations:** Research Institute of General Surgery, Jinling Hospital, Medical School of Nanjing University, Nanjing, Jiangsu China; Department of General Surgery, Jinling Hospital, South Medical University, Guangdong, China

## Abstract

**Introduction:**

Guidelines support the use of enteral nutrition to improve clinical outcomes in critical illness; however, the optimal calorie and protein intake remains unclear. The purpose of this meta-analysis was to quantitatively analyze randomised controlled trials with regard to clinical outcomes related to varying calorie and protein administration in critically ill adult patients.

**Method:**

We searched Medline, EMBASE, and Cochrane databases to identify randomised controlled trials that compared the effects of initially different calorie and protein intake in critical illness. The risk ratio (RR) and weighted mean difference with 95% confidence intervals (CI) were calculated using random-effects models. The primary endpoint was mortality; secondary endpoints included infection, pneumonia, gastrointestinal intolerance, hospital and intensive care unit lengths of stay, and mechanical ventilation days.

**Results:**

In the eight randomised controlled trials that enrolled 1,895 patients there was no statistical difference between the low-energy and high-energy groups in mortality (RR, 0.90; 95% CI, 0.71 to 1.15; *P* = 0.40), infection (RR, 1.09; 95% CI, 0.92 to 1.29; *P* = 0.32), or the risk of gastrointestinal intolerance (RR, 0.84; 95% CI, 0.59 to 1.19; *P* = 0.33). In subgroup analysis, the low-energy subgroup, fed 33.3 to 66.6% of goal energy, showed a lower mortality than the high-energy group (RR, 0.68; 95% CI, 0.51 to 0.92; *P* = 0.01). The improvements in mortality and gastrointestinal intolerance were absent when calorie intake was >66.6% of goal energy in the low-energy group. High-energy intake combined with high-protein intake reduced the infections (RR, 1.25; 95% CI, 1.04 to 1.52; *P* = 0.02); however, when the daily protein intake was similar in both groups, a high-energy intake did not decrease the infections. No statistical differences were observed in other secondary outcomes.

**Conclusion:**

This meta-analysis indicates that high-energy intake does not improve outcomes and may increase complications in critically ill patients who are not malnourished. Initial moderate nutrient intake (33.3 to 66.6% of goal energy), compared to high energy, may reduce mortality, and a higher protein intake combined with high energy (≥0.85 g/kg per day) may decrease the infection rate. However, the contribution of energy versus protein intake to outcomes remains unknown.

**Electronic supplementary material:**

The online version of this article (doi:10.1186/s13054-015-0902-0) contains supplementary material, which is available to authorized users.

## Introduction

Enteral nutrition (EN) may not only supply nutrition to patients but also protect intestinal epithelial cells, improve intestinal tight junctions, support intestinal structure and function, and prevent bacterial translocation [1-5]. However, no such beneficial clinical effect of EN has been observed in the first large randomised controlled trial (RCT) comparing isocaloric EN to parenteral nutrition (PN) [5]. Critically ill patients are often in a catabolic state because of the influence of inflammatory cytokines and stress hormones [6]. As a result, patients in the ICU may be at an increased risk of progressive underfeeding, which can result in malnutrition. To avoid malnutrition, the guidelines of several health organisations advocate early EN [7-10]. Unfortunately, critically ill patients may not receive sufficient calories and protein via EN because of gastrointestinal dysmotility, particularly delayed gastric emptying [11]. Initial feeding up to the caloric target may lead to high gastric residual volumes, which may increase the risk of aspiration pneumonia and result in longer ICU stays and increased mortality [5,12]. However, a limited-energy supplement that is provided because of gastrointestinal intolerance can increase the risk of malnutrition, which may be associated with impaired immune function, weakened or wasted muscles, increased duration of mechanical ventilation, and infectious complications [13,14]. Therefore, the initial optimal calorie and protein recommendations for critically ill patients are still unclear.

The European Society for Clinical Nutrition and Metabolism’s guidelines suggest that an exogenous energy supply in excess of 20 to 25 kcal/kg body weight per day may be associated with less favourable outcomes during the acute phase of a critical illness (grade C) [10]. Similarly, the American Society for Parenteral and Enteral Nutrition reported that providing >50% to 65% of goal calories should be attempted as a means of achieving the clinical benefit of EN during the first week of hospitalisation (grade C) [4,7]. However, the Canadian Critical Care Nutrition Clinical Practice Guidelines, because of insufficient data, did not recommend using hypocaloric EN in critically ill patients [9]. Because these three guidelines were published before 2014, when three relevant RCTs were published, the summary of evidence for EN in critical illness requires updating. Furthermore, the level of evidence of these recent RCTs has not been evaluated.

A cohort study of patients in a respiratory ICU reported that inadequate calorie delivery is associated with higher odds of mortality [15]. Surprisingly, a study comparing hypocaloric and normocaloric nutrition in critically ill patients demonstrated that initial hypocaloric feeding did not affect the hospital or ICU mortality [16]. Conflicting results have even been observed within the same study; the Tight Calorie Control Study showed that near-target energy intake was associated with lower hospital mortality in the per protocol analysis, but increased the duration of the ICU stay and rate of infectious complications in the intention to treat analysis [17]. Therefore, given the heterogeneity of these studies, a meta-analysis of more recent RCTs is needed. The current meta-analysis of eight RCTs aimed to compare initial hypocaloric EN versus hypercaloric EN, with different protein intakes, in critically ill patients.

## Methods

### Search strategy

In this systematic review, we conducted a search of the published literature to identify all relevant clinical trials using the keywords or MeSH headings of ‘trophic feeding’, ‘hypocaloric nutrition’, ‘permissive underfeeding’, ‘gradual enteral nutrition’, ‘standard enteral nutrition’, ‘intensive enteral nutrition’, ‘concentrated enteral nutrition’, ‘hypercaloric nutrition’, ‘normocaloric nutrition’, ‘full feeding’, ‘critical illness’, ‘critically ill’, ‘ICU’, ‘acute lung injury’, ‘respiratory insufficiency’, and ‘intensive care’ in combination with the Boolean operators AND and OR. Two of the authors (Feng Tian and Xinying Wang) independently performed computerised searches in the MEDLINE, EMBASE, and Cochrane Controlled Trials Register databases. We also searched previous review articles for additional original studies. The final search was conducted on 1 November 2014. No language restrictions were included in the searches. Additionally, abstracts from major scientific meetings were included if they were available for data extraction; authors were approached for additional or missing data, if necessary.

### Inclusion criteria

Two investigators (Feng Tian and Xuejin Gao) independently reviewed all original studies to determine inclusion or exclusion; in the case of disagreement, a third author was consulted. Original studies were selected for inclusion if they met the following criteria: 1) the research design was an RCT or randomized trial or study; 2) the population comprised critically ill adult patients admitted to the ICU (>16 years old); 3) the intervention of the study was designed such that the two groups received significantly different calorie intakes by EN; and 4) the clinical outcome of overall mortality of critically ill patients was reported.

### Exclusion criteria

Studies were excluded if they met either of the following criteria: 1) the target calorie supply was different between the two groups; and 2) EN was administered for <2 days.

### Data extraction

Two authors (Feng Tian and Xuejin Gao) independently extracted the following variables where they were published and available: demographics, sample size, critical illness severity, amount of daily calorie and protein intake, body mass index (BMI), intervention provided in both groups, and clinical outcomes.

### Assessment of quality and risk of bias

The same two authors independently assessed the study quality and risk of bias using the methods detailed in the Cochrane Handbook for Systematic Reviews of Interventions [18]. Disagreements were resolved in pairs of authors by consensus. The six quality criteria were random sequence generation, allocation concealment, blinding of participants and personnel, blinding of outcome assessment, incomplete outcome data, and selective reporting.

### Statistical analysis

The primary outcome of this meta-analysis was overall mortality. Hospital mortality in all studies was combined. If hospital mortality was not reported, 60- or 90-day or ICU mortality was used instead. Secondary outcomes included infections (bacteraemia or sepsis was used if the number of infection cases was not reported), pneumonia (ventilator-associated pneumonia and infectious pneumonia), length of stay (LOS) in both hospital (LOS-HOS) and ICU (ICU LOS), and mechanical ventilation days (MVD); these were also pooled for all studies. Despite differences in the definition of gastrointestinal intolerance (high residual gastric volume, regurgitation, vomiting, noninfectious diarrhoea, or abdominal distension), we combined the outcomes where diarrhoea was reported.

The risk ratio (RR) and associated 95% confidence interval (CI) were used to summarize binary outcomes including mortality, infections, and pneumonia. The overall weighted mean difference (WMD) with 95% CI was estimated for LOS and MVD. A more conservative random-effects model was used in this meta-analysis because of anticipated heterogeneity. Statistical heterogeneity among trials is expressed as the *P* value (Cochran’s Q statistic), where a *P* < 0.05 and I^2^ statistic >50% indicated significant heterogeneity. Subgroup analyses were conducted to find the source of heterogeneity, and a test for heterogeneity interaction was performed to determine whether there was a difference in effect sizes between subgroups. The subgroups were chosen based on their suspected influences on the analyses and previous relevant studies. For instance, the varying calorie intake between studies could influence the aggregate results. Therefore, the eight studies were divided into three subgroups according to the percentage of target energy achieved in the low-energy (LE) group (<33.3%, 33.3 to 66.6%, and >66.6%). The cut-offs of 33.3% and 66.6% were based on a previous cohort study and a recent meta-analysis [19,20]. The overlap in studies was managed with subgroup analysis. Funnel plots were used to assess possible bias in reporting and publication; forest plots are presented as pooled data with Review Manager, version 5.3 (Copenhagen: The Nordic Cochrane Centre, The Cochrane Collaboration, 2014).

## Results

### Results of the literature search and study selection

**Figure 1:**
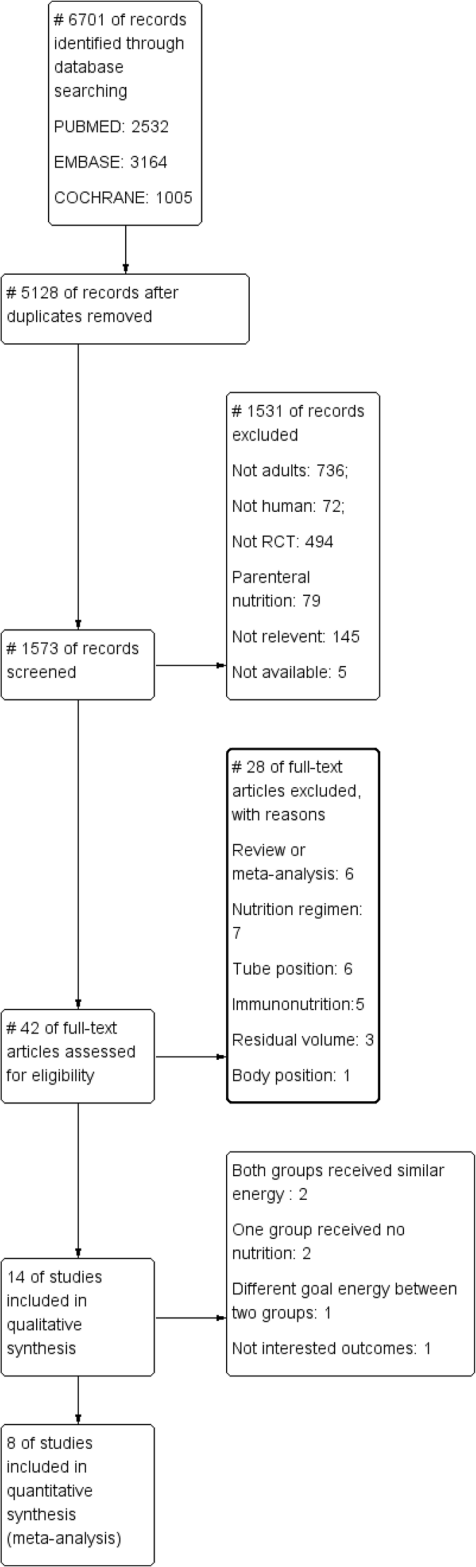
Preferred Reporting Items for Systematic Reviews and Meta-Analyses (PRISMA) statement and accompanying study flow diagram. RCT, randomised controlled trial.

Our detailed search strategy is illustrated in the flow diagram in Figure 1, which follows the strategy recommended by the Preferred Reporting Items for Systematic Reviews and Meta-Analyses group. Our initial search of the electronic databases yielded 6,701 articles: 5,128 articles were duplicates; 808 articles did not include adult patients; 494 articles were not RCTs; 79 articles were of PN; 145 articles were not relevant; five articles were not available; six articles were reviews or meta-analyses; seven articles included nutrition regimens; six articles were conducted for tube position; five articles were related to immunonutrition; three articles involved residual volume; and one article described body position. Of the 14 potentially relevant articles that were selected for further assessment, six were excluded: two studies reported that both groups were similar, two studies did not administer EN to the LE group, one reported different goal energy levels between the two groups, and one study reported blood glucose and albumin as outcomes, rather than mortality. Thus, eight studies published between 1999 and 2014 fulfilled the inclusion criteria; as a result, 1,895 patients were included in this meta-analysis, of which 951 were in the LE group and 944 were in the high-energy (HE) group [21-28].

### Risk of bias in included studies

**Figure 2:**
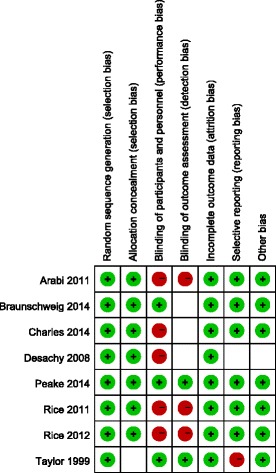
Risk of bias summary: authors’ judgments regarding the risk of bias factor for each included study.

**Figure 3:**
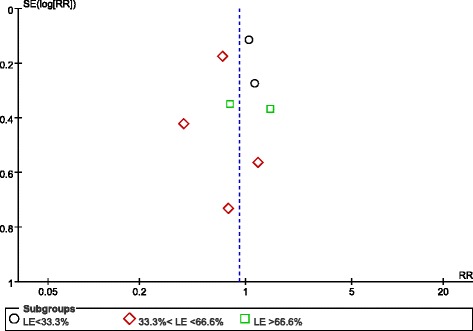
Funnel plots showing the impact of initial low energy intake on mortality. LE, low-energy; RR, risk ratio; SE, standard error.

**Figure 4:**
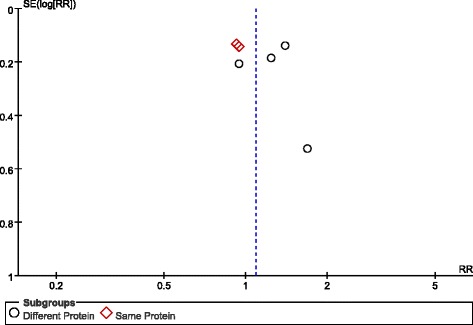
Funnel plots showing the impact of initial low energy intake on infections. RR, risk ratio; SE, standard error.

**Figure 5:**
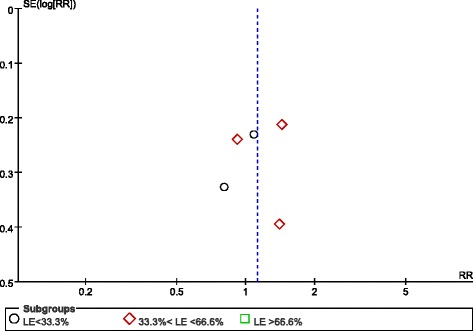
Funnel plots showing the impact of initial low energy intake on pneumonia. LE, low-energy; RR, risk ratio; SE, standard error.

Randomisation methods were reported in all eight studies. Allocation concealment was used in six trials using sealed envelopes [21-26], and, although one study did not state allocation concealment, we agreed that this study’s allocation method was low risk for our purposes because it used a web-based randomization system [27]. Because of different nutrition doses and titration needs for tolerance and gastric residuals, only two studies used a double-blind design [25,28]. Protocol violations after randomisation occurred in four studies [21,23,25,27]. The assessment for risk of bias in each study is shown in Figure 2. When more than four studies were included, funnel plots were used to assess possible reporting or publication bias (Figures 3, 4 and 5). Figures 3 and 5 show the approximate symmetry of the funnel plots for mortality and pneumonia. Figure 4 shows the asymmetry of the funnel plots for infection; in light of this, we performed a sensitivity analysis to evaluate the robustness of the pooled outcome.

### Characteristics of the included studies

The basic characteristics of patients in the included studies are shown in Tables 1 and 2. The mean age of patients was >50 years, except in one study whose patients were <35 years of age [28]. The intervention duration in five studies was 5 to 7 days [21,24,26-28] and >10 days in three studies [22,23,25]. The Acute Physiology and Chronic Health Evaluation (APACHE) II scores in four studies were >20 [21,22,25,26] and <20 in two studies [23,28]. One study used the Simplified Acute Physiology Score [24], and another study used APACHE III scores [27]. The mean BMI of patients in seven studies was >25 kg/m^2^ [21-27]; one study did not report BMI [28].

**Table 1:** **Characteristics of the enrolled studies**

**Study**	**No. randomized**		**Characteristics of patients**	**BMI (SD)**		**Mean APACHE II score (SD)**	
	**LE**	**HE**		**LE**	**HE**	**LE**	**HE**
Arabi *et al*. [21]	120	120	>18 years old	28.5 (7.4)	28.5 (8.4)	25.2 (7.5)	25.3 (8.2)
			ICU >48 hours				
Braunschweig *et al*. [22]	38	40	>18 years old	30.1 (8.9)	29.8 (9.3)	27.7 (7.9)	23.4 (9.3)
			Medical or surgical ICU				
			ALI <24 hours				
Charles *et al*. [23]	41	42	>18 years old	32.9 (2.0)	28.1 (0.9)	16.6 (0.9)	17.3 (0.8)
			Artificial nutrition >48 hours				
			ICU >48 hours				
Desachy *et al*. [24]	50	50	>18 years old	27 (5)	25 (3)	40 (11)^1^	42 (17)^1^
			Medical or surgical ICU				
			Mechanical ventilation >72 hours				
Peake *et al*. [25]	55	57	≥18 years old	26.2 (6.4)	27.8 (7.9)	22 (8.9)	23 (9.1)
			Enteral nutrition ≥2 days				
			Mechanical ventilation				
Rice *et al*. [26]	98	102	ICU admission	29.2 (10.2)	28.2 (9.4)	26.9 (8.1)	26.9 (6.6)
			Mechanical ventilation >72 hours				
Rice *et al*. [27]	508	492	ICU admission	29.9 (7.8)	30.4 (8.2)	92 (28)^2^	90 (27)^2^
			ALI <48 hours				
			Mechanical ventilation >72 hours				
Taylor *et al*. [28]	41	41	Head injury necessitating mechanical ventilation	N/A	N/A	14^3^	14^3^
			Glasgow Coma Scale >3				

^1^Mean APACHE III score. ^2^Mean Simplified Acute Physiology Score II. ^3^Standard deviation was not reported. ALI, acute lung injury; APACHE, Acute Physiology And Chronic Health Evaluation; BMI, body mass index; HE, high-energy; LE, low-energy; N/A, not available; SD, standard deviation.

**Table 2:** **Nutritional targets and interventions**

**Study**	**Duration of study (days)**	**Daily target calorie supply (Kcal/kg)**		**Mean daily percentage of caloric goal (%)**		**Mean daily protein intake (g/kg)**	
		**LE**	**HE**	**LE**	**HE**	**LE**	**HE**
Arabi *et al*. [21]	7	N/A^1^	N/A^1^	59.0	71.4	65.2%^2^	63.7%^2^
Braunschweig *et al*. [22]	20	25-30	25-30	55.4	84.7	0.68	0.95
Charles *et al*. [23]	10-12	25-30	25-30	40.5	73	1.1	1.1
Desachy *et al*. [24]	5	25	25	76	95	N/A	N/A
Peake *et al*. [25]	10	25-30	25-30	72	102	1.05	1.02
Rice *et al*. [26]	7	25-30	25-30	15	74.8	0.17	0.85
Rice *et al*. [27]	6	25-30	25-30	25	80	N/A	N/A
Taylor *et al*. [28]	7	35	35	36.8	59.2	0.57	1.03

^1^The caloric requirement was estimated using the Harris-Benedict equation and adjusted for stress factors. ^2^The percentage of protein requirement was calculated on the basis of patient condition and underlying diseases. HE, high-energy; LE, low-energy; N/A, not available.

The mean daily delivered caloric and protein levels differed among studies. In the LE group, the mean daily percentage of target calories in two studies was <33.3%; in four studies, it was between 33.3% and 66.6%; and in two studies, it was >66.6%. In the HE group, the percentage of target calories for patients was >70% in seven studies and >90% in two studies, but it was 59.2% in one study. Although the caloric intake was 59.2% for the HE group in the study by Taylor and colleagues [28], the mean target calorie intake was 35 kcal/kg per day. Therefore, the actual energy intake in the HE group was 20.72 kcal/kg per day (in the LE group it was 12.88 kcal/kg per day), which was similar to the caloric intake of the HE group in the other studies. Among the six studies [21-23,25,26,28] that reported daily protein intake levels, the protein levels were different between the HE and LE groups in three studies [22,26,28]. In one study, the protein intake was not reported clearly; however, based on the author’s reply that the use of different enteral formulas led to different protein amounts, the different levels of protein intake for the two groups could be estimated [27]. In the studies in the different-protein subgroup, the daily protein intake was ≥0.85 g/kg per day in each HE group and ≤0.68 g/kg per day in each LE group, when adjusted by ideal body weight.

### Meta-analysis of primary outcomes

**Figure 6:**
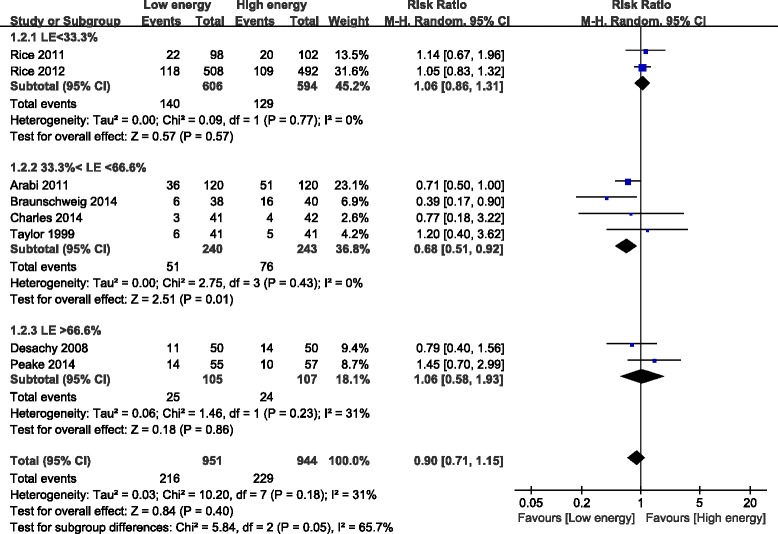
Impact of initial low energy intake on mortality. CI, confidence interval; LE, low energy; MH, Mantel-Haenszel.

The primary meta-analysis included all eight studies (1,895 participants) and revealed that mortality was not significantly different for patients in the LE group compared with the HE group (RR, 0.90; 95% CI, 0.71 to 1.15; *P* = 0.40; I^2^ = 31%; *P* = 0.18). Subgroup analysis was performed according to the percentage of the goal energy achieved and showed that mortality was significantly different among the three subgroups. Compared with the HE group, mortality was significantly lower in the LE group (fed between 33.3% and 66.6% of the goal energy in the LE group; RR, 0.68; 95% CI, 0.51 to 0.92; *P* = 0.01; I^2^ = 0%; *P* = 0.43). In contrast, compared with the HE group, mortality was not different in the LE subgroup that was fed <33.3% (RR, 1.06; 95% CI, 0.86 to 1.31; *P* = 0.57; I^2^ = 0%; *P* = 0.77) or >66.6% of the goal energy (RR, 1.06; 95% CI, 0.58 to 1.93; *P* = 0.86; I^2^ = 31%; *P* = 0.23). There was significant heterogeneity among the subgroups (I^2^ = 65.7%; *P* = 0.05) (Figure 6). Subgroup analysis was also performed based on the differences in daily protein intake between the two groups (studies with different protein levels were in one subgroup and studies with the same protein levels were in another subgroup) and showed that mortality was not different between the LE and HE groups in the different or same protein subgroup (I^2^ = 0%; *P* = 0.87) (see Additional file 1).

### Meta-analysis of secondary outcomes

#### Infectious complications

Six of the eight studies reported infections; these studies included 1,683 patients [21-23,26-28]. The meta-analysis showed no significant difference in infectious complications between the LE and HE groups (RR, 1.09; 95% CI, 0.92 to 1.29; *P* = 0.32; I^2^ = 31%; *P* = 0.20).

**Figure 7:**
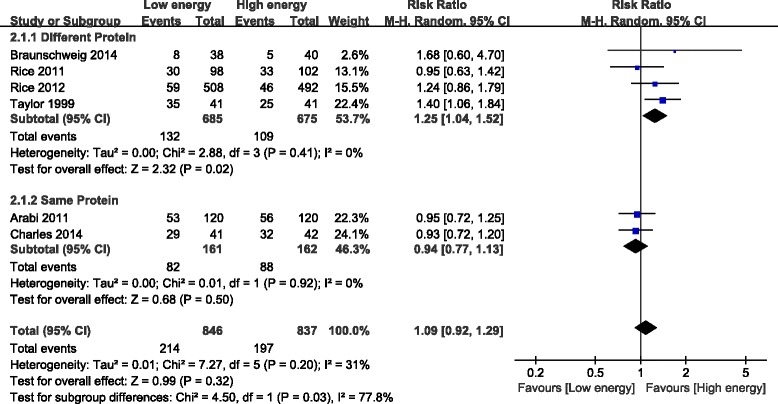
Impact of initial low energy intake on infections. CI, confidence interval; MH, Mantel-Haenszel.

Subgroup analysis was performed based on daily protein intake differences between the two groups. Relatively higher daily protein and calorie intake decreased infection in the different protein intake subgroups (RR, 1.25; 95% CI, 1.04 to 1.52; *P* = 0.02; I^2^ = 0%; *P* = 0.41). Interestingly, when the daily protein intake was similar, infectious complications were not different between the LE and HE groups (RR, 0.94; 95% CI, 0.77 to 1.13; *P* = 0.50; I^2^ = 0%; *P* = 0.92). There was significant heterogeneity among the subgroups (I^2^ = 77.8%; *P* = 0.03) (Figure 7).

#### Pneumonia

**Figure 8:**
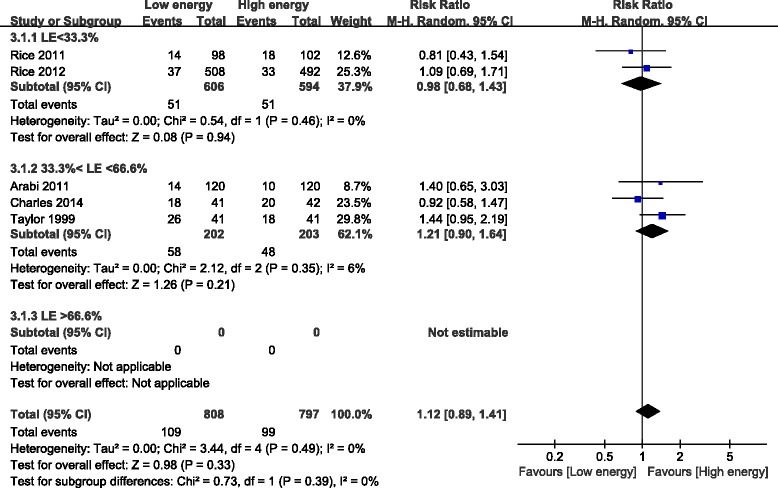
Impact of initial low energy intake on pneumonia. CI, confidence interval; LE, low energy; MH, Mantel-Haenszel.

Five of the eight studies reported infections; these studies included 1,605 patients [21,23,26-28]. Pneumonia was not significantly different between the groups (RR, 1.12; 95% CI, 0.89 to 1.41; *P* = 0.33; I^2^ = 0%; *P* = 0.49). Subgroup analysis showed that pneumonia was not affected by the percentage of the goal energy achieved (I^2^ = 0%; *P* = 0.39) or the daily protein intake (I^2^ = 0%; *P* = 0.67) (Figure 8).

#### Gastrointestinal intolerance

**Figure 9:**
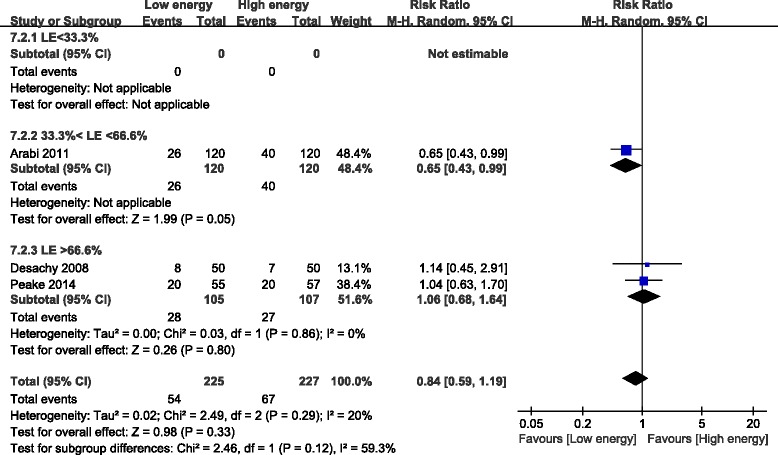
Impact of initial low energy intake on gastrointestinal intolerance. CI, confidence interval; LE, low energy; MH, Mantel-Haenszel.

Three of the eight studies reported gastrointestinal intolerance; these studies included 452 patients [21,24,25]. Patients initially receiving LE did not decrease the risk of gastrointestinal intolerance compared with those initially receiving HE (RR, 0.84; 95% CI, 0.59 to 1.19; *P* = 0.33; I^2^ = 20%; *P* = 0.29). Subgroup analysis showed that gastrointestinal intolerance in the LE group that was fed 33.3% to 66.6% of the goal energy was significantly lower than that in the HE group (RR, 0.65; 95% CI, 0.43 to 0.99; *P* = 0.05). However, this advantage was not obvious in the LE group that was fed >66.6% of the goal energy (RR, 1.06; 95% CI, 0.68 to 1.64; *P* = 0.80; I^2^ = 0%; *P* = 0.86), compared with the HE group fed >95% of the goal energy (Figure 9).

#### Length of intensive care unit stay

The data from four studies with 501 patients showed no statistically significant differences for ICU LOS between the two groups (WMD, −0.50; 95% CI, −2.99 to 1.99; *P* = 0.69; I^2^ = 22%; *P* = 0.28) [21-24]. Subgroup analysis indicated that the LOS-HOS was not affected by the percentage of the goal energy achieved (I^2^ = 0%; *P* = 0.91) or daily protein intake (I^2^ = 0%; *P* = 0.97) (see Additional file 2).

#### Length of hospital stay

On the basis of the meta-analysis of four studies with 501 participants, the LOS-HOS was not significantly different between the groups (WMD, −1.64; 95% CI, −7.35 to 4.07; *P* = 0.57; I^2^ = 0%; *P* = 0.60) [21-24]. Subgroup analysis indicated that LOS-HOS was not affected by the percentage of the goal energy achieved (I^2^ = 0%; *P* = 0.80) or daily protein intake (I^2^ = 0%; *P* = 0.39) (see Additional file 3).

#### Mechanical ventilation days

**Figure 10:**
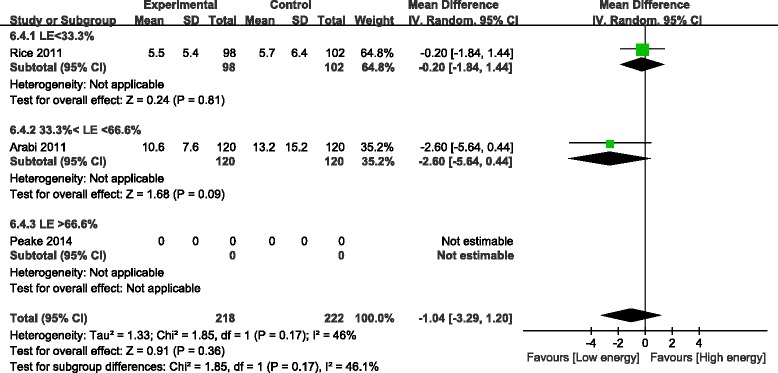
Impact of initial low energy intake on mechanical ventilation days. CI, confidence interval; IV, inverse variance; LE, low energy.

For the two studies of 440 patients that provided data on MVD, our meta-analysis showed no difference between the LE and HE groups (WMD, −1.04; 95% CI, −3.29 to 1.20; *P* = 0.36; I^2^ = 46%; *P* = 0.17) [21,26] (Figure 10).

## Discussion

In this meta-analysis of eight RCTs of critically ill adult patients, there were no statistically significant differences in mortality, infections, pneumonia, ICU LOS, LOS-HOS, MVD or gastrointestinal intolerance between the LE and HE groups. In the subgroup analysis of the LE group that was fed between 33.3% and 66.6% of the goal energy, mortality was significantly lower than in the HE group. Nevertheless, when the LE group was fed <33.3% or >66.6% of the goal energy, this advantage in mortality was not present, nor was the decreased risk for gastrointestinal intolerance, compared with the HE group. Furthermore, when the HE group received more protein than the LE group, infections decreased. This advantage was no longer present when the patients received similar protein levels; however, these findings should be confirmed with additional studies, given the methodological limitations of the present study as described later in the discussion.

Current guidelines recommend that nutrition should be provided to critically ill adults owing to the risk of malnutrition. In addition, nutrition should be initiated as soon as possible, ideally within 24 to 48 hours of ICU admission [4,7,9]. Early EN improves outcomes in critical illness and may be the optimal delivery route in those patients with adequate gastrointestinal tract function, compared with PN [5,7]. However, weak gastrointestinal function limits the effective delivery of EN. For more effective delivery, supplemental parenteral nutrition (SPN) was recently attempted; however, consistent results were not obtained from different trials regarding whether SPN results in harm, and no study showed clear benefit [29]. Therefore, consensus regarding the early use of SPN as a way to supply sufficient energy to patients does not exist [30-32]. Although PN may have resulted in the negative outcomes in these studies, recent RCTs have shown that PN does not harm the critically ill [5]. Owing to the conflicting results, the calorie dose is suspected as important for clinical outcomes in critically ill patients [5,33,34].

Since 2009, several cohort studies have investigated the relationship between calorie intake and clinical outcomes in critical illness. A multicentre cohort study of 2,772 patients in 2009 indicated that increased energy and protein intake might be associated with reduced mortality and MVD [35]. However, another cohort study suggested that reduced caloric intake improves clinical outcomes in critically ill patients [36]. The nutrition regimen in these studies involved EN, PN, and EN with PN. Because the benefits of EN might be counteracted by other nutrition regimens, the present meta-analysis focused on calorie and protein intake using EN alone.

The recent major guidelines on EN in critically ill patients are not supported by our results [9,10], perhaps because those recommendations were based on only two previous RCTs [28,37]. However, more relevant studies, including three related RCTs in the past year, have been reported since the guidelines were published. The recent meta-analysis by Choi and colleagues [20] of four RCTs regarding the relationship between clinical outcomes and EN-administered calories in critically ill adults showed that overall mortality was significantly reduced when underfed patients received ≥33.3% of the standard caloric requirement; however, differences between subgroups of 33.3 to 66.6% and >66.6% standard caloric requirements were not investigated because of the low number of included studies. This is despite the results of a previous cohort study that demonstrated that patients in the middle tertile (33 to 65% of target energy) were more likely to be discharged from the ICU with spontaneous ventilation than patients in the lowest and highest tertiles [19]. The risk of gastrointestinal intolerance was higher with increased energy supplied by EN; in turn, mortality increased, which might support the benefits of LE [12]. Similarly, in the present meta-analysis, mortality was similar in the LE group who were fed >66.6% of the goal energy compared with the HE group.

In addition, we found that decreased infection rates might be attributed to HE combined with high protein intake, which is consistent with previous observational studies that declared that a relatively higher protein intake might improve clinical outcomes [38,39]. Because lower infection rates were observed with daily protein intake ≥0.85 g/kg, compared with ≤0.68 g/kg, daily protein intake ≥0.85 g/kg might be effective for prevention of infections, despite being lower than recommendations (1.2 to 1.5 g/kg per day). Moreover, the finding that infections did not increase mortality is noteworthy. It is possible that mortality could not be determined by infections alone; for example, some infections may not be serious enough to result in death. In the study by Taylor and colleagues, infections were significantly higher in the LE group without any differences in mortality between the LE and HE groups [28]. Similarly, in the study by Braunschweig and colleagues [22], a dramatic increase in mortality was observed in the HE group despite relatively lower infection rates. That was possibly explained by the increased mortality as patients who die in ICU no longer develop infections. Furthermore, infectious outcomes are more vulnerable to bias in these often open-label studies, which might also explain the isolated finding of fewer infectious diagnoses but no effect on clinical outcome. However, in the present study, no specific search was conducted for low versus high protein intake, and related RCTs for LE combined with high protein intake were lacking. Moreover, we were not able to explore other aspects, such as pneumonia or LOS-HOS, because of the limited number of studies and the diverse protein dose among studies. Therefore, we could not provide an optimal range of daily protein intake, but we noted that, as protein intake increased, the infection rate decreased. More studies using similar protocols are required to determine optimal intake levels. In addition, none of the eight included studies showed that patients were administrated the recommended amount of protein, suggesting that protein deficiency might not be appropriately addressed in clinical practice and daily protein deficiency might impact clinical outcomes, such as infections. More concern should be given to protein supply in the critically ill patients in the future.

Although we attempted to reduce bias, the results of the present study should be treated with caution because of certain limitations. First, this meta-analysis included a small number of studies; although we included four studies more than a previous meta-analysis [20], data regarding gastrointestinal intolerance in the subgroup that received <33.3% of the goal energy are lacking. Thus, more high-quality studies are needed before a reliable conclusion can be determined. Second, the disease severity reported by the studies differed. The low APACHE II scores in two studies were associated with low mortality, which might have contributed to heterogeneity [23,28]. Third, the significant variations in calorie intake in the LE groups might have resulted in significant overlap between studies, potentially affecting the aggregated results. We attempted to overcome this variability and potential overlap through subgroup analysis, but the results should still be treated with caution. Fourth, the data were presented differently between studies; for instance, four studies presented the median MVD rather than mean MVD [22,23,25,27]. Because we did not believe these should be used together in our meta-analysis, only two studies were included in the MVD analysis. For the same reason, only four of eight studies were included in the ICU LOS and LOS-HOS analyses, and only one of the four studies was in the different-protein subgroup, while the other three studies were in the same-protein subgroup. As a result, the ICU LOS and HOS-LOS in the HE with high protein subgroup was only present in the study by Braunschweig and colleagues rather than in the aggregated results of four studies, and the results could not accurately reflect whether HE with high protein influences the LOS. Within the three studies of the same-protein subgroup, the ICU LOS and LOS-HOS were not different between the HE and LE groups. Therefore, the pooled results could only suggest that no effects were present in the comparison of the HE and LE groups. Fifth, the intervention duration in three studies was longer than in the other five studies; however, the results did not vary significantly by subgroup analysis. Sixth, none of the selected studies, except one, stated that the mean BMI of patients was >25 kg/m^2^, which suggests that our conclusions may not be appropriate for malnourished patients. In addition, although the sensitivity analysis revealed that the overall pooled results were stable, the conclusions for the subgroups were affected; therefore, the results should be interpreted with caution, and additional studies are required.

## Conclusions

In summary, there were no significant differences between the initial LE and HE groups of critically ill adult patients. According to this meta-analysis, initial moderate nutrient intake (33.3% to 66.6% of goal energy) as compared to HE may reduce mortality, and a higher protein intake combined with HE (≥0.85 g/kg per day) may decrease the infection rates. However, because of the study limitations, the optimal dose of initial calorie and protein intake is still uncertain, and further large-sample RCTs are needed to confirm our conclusions.

## Key messages

- Initial moderately low calorie intake could improve the prognosis of critical illness and excessively low or high calorie intake should be avoided in these patients.
- A relatively high protein intake might benefit critically ill patients, but the daily administration of protein should not exceed the upper limit of the recommended range.
- The current data are not sufficient to draw conclusions regarding the optimal initial calorie and protein intake to be administered by EN in non-malnourished critically ill adults because of the limited number and heterogeneity of the trials, including the varying calorie and protein intake in the eight included studies.
- More rigorously designed studies using similar protocols are required to determine the optimal levels of calorie and protein intake.

## Additional files

Additional file 1:
**Impact of initial low energy intake on mortality (subgroup analysis of protein intake).** CI, confidence interval; MH, Mantel-Haenszel. Additional file 2:
**Impact of initial low energy intake on length of intensive care unit stay.** CI, confidence interval; IV, inverse variance; LE, low energy. Additional file 3:
**Impact of initial low energy intake on length of hospital stay.** CI, confidence interval; IV, inverse variance; LE, low energy.

## Abbreviations

APACHE: Acute Physiology and Chronic Health Evaluation
BMI: body mass index
CI: confidence interval
EN: enteral nutrition
HE: high-energy
ICU LOS: intensive care unit length of stay
LE: low-energy
LOS: length of stay
LOS-HOS: hospital length of stay
MVD: mechanical ventilation days
PN: parenteral nutrition
RCT: randomised controlled trial
RR: risk ratio
SPN: supplemental parenteral nutrition
WMD: weighted mean difference
